# Postpartum hemorrhage: prevention, diagnosis and non-surgical management: Number 5 - November 2020

**DOI:** 10.1055/s-0040-1721882

**Published:** 2020-11-30

**Authors:** Álvaro Luiz Lage Alves, Adriana Amorim Francisco, Gabriel Costa Osanan, Laíses Braga Vieira

**Affiliations:** 1 Faculdade Ciências Médicas de Minas Gerais, Belo Horizonte, MG, Brazil; 2 Faculdade de Enfermagem, Universidade Federal de São Paulo, São Paulo, SP, Brazil; 3 Faculdade de Medicina, Universidade Federal de Minas Gerais, Belo Horizonte, MG, Brazil; 4 Hospital das Clínicas Gaspar Vianna, Belém, PA, Brazil

## Key points

- Postpartum hemorrhage is the world's leading cause of maternal death and peripartum hysterectomy.
- The main causes of postpartum hemorrhage are uterine atony, birth canal trauma, retention of placental remains and coagulation disorders.
- Risk stratification for postpartum hemorrhage optimizes care planning and promotes early adoption of preventive measures.
- Bleeding control within the first hour of diagnosis (“golden hour”) is the most effective measure for treating postpartum hemorrhage.
- The shock index is the clinical method of choice for estimating blood loss and a good parameter to guide the need for blood transfusion.
- The main drugs used in pharmacological therapy for postpartum hemorrhage are oxytocin, ergot derivatives, misoprostol and tranexamic acid.
- In uterine atony with pharmacological therapy failure, the intrauterine balloon tamponade should precede the surgical approach.
- The non-pneumatic anti-shock garment is useful in postpartum hemorrhage with hemodynamic instability and enables continuity of treatment and patient transfers.

## Recommendations

- Every pregnant woman with a previous cesarean section should have an ultrasound scan for placental location. In case of placenta previa, placental dopplerfluxometry and investigation of other ultrasound signs of placenta accreta are indicated. Faced with the suspicion of parametrial invasion and in the placenta previa located on posterior wall, nuclear magnetic resonance or three-dimensional ultrasound may contribute to the investigation. The delivery of these pregnant women must take place in a tertiary service.
- The main preventive measure for postpartum hemorrhage is the intramuscular administration of 10 units of oxytocin immediately after birth, associated with active management of the third stage.
- The sequencing of care in postpartum hemorrhage should include requesting help, performing a uterine compression maneuver, rapid assessment of etiology, maintaining oxygenation and tissue perfusion, obtaining large venous accesses with blood sample collection and request for laboratory tests, blood volume replacement, administration of tranexamic acid and uterotonics, evaluation of antibiotic prophylaxis and blood loss estimation.
- Blood loss can be estimated by visual assessment, weighing of surgical compresses, use of collecting devices or by clinical methods.
- Volume resuscitation with crystalloids should not exceed 2,000 mL and transfusion of blood components is indicated for hypovolemic shock, especially if moderate or severe. Hemodynamically unstable patients with significant blood loss should receive emergency transfusion of two red cell concentrates. If crossmatching is not available, O negative blood should be transfused.
- The intrauterine balloon tamponade can be employed after vaginal delivery and during or after cesarean section with specific volumes of infusion. Depending on the tamponade test, balloons with drainage function should be preferred. Uterotonics and antibiotics should be administered during the entire tamponade time. The balloon should be removed after hemodynamic stability, through deflation in stages and with a reserved operating room.

## Clinical context

Postpartum haemorrhage (PPH) is defined as the cumulative blood loss of 1,000 mL or more, accompanied by signs or symptoms of hypovolemia within 24 hours after birth. 1 Currently, this is the leading cause of maternal death worldwide, with about 140,000 deaths annually and the frequency of one death every four minutes. 2 Most of these deaths are considered preventable and occur in low- and middle-income countries. 3

In addition to high mortality, a significant number of patients who survive severe PPH evolve with physical and/or emotional sequelae. 4 Therefore, it is essential that all institutions and professionals that provide childbirth care are properly prepared to prevent, diagnose and manage a condition of PPH.

## What is the golden hour in PPH?

Early control of the bleeding site is the most effective strategy for preventing hypovolemic shock. The term “golden hour in obstetrics” has been introduced in this context, referring to a strategy for controlling the hemorrhagic site within the first hour after its diagnosis. 5 6 Note that massive bleeding requires even earlier control in order to avoid serious maternal complications. Through an early, aggressive, efficient, organized approach without delay, the deadly triad of hemorrhagic shock (hypothermia, acidosis and coagulopathy) can be avoided. 6 To this end, it is essential that the golden hour is linked to the presence of a warning and response system for PPH.

## What are the characteristics of an Obstetric Warning and Response System (OWRS) for PPH?

The obstetric warning and response system is an orderly work system aimed at organizing and coordinating actions to reduce the risk and morbidity and mortality of PPH. 7 8

The OWRS proposes the implementation of work processes that include risk stratification of PPH, routine and universal use of uterotonics after births, timely diagnosis of cases and continuous monitoring of patients. It is essential to organize care flows with well-defined massive management and transfusion protocols and availability of PPH kits accessible to the entire care team in order to ensure a safe and effective service. The OWRS promotes the involvement and training of multidisciplinary and intersectoral teams and encourages the implementation of a communication and learning system, thereby helping to develop constructive leadership skills and allowing continuous monitoring of risk situations. It also contributes to the organization of the health network with the aim to guarantee care flows, access to more complex treatments and care transfers. 9

## What are the preventive measures in PPH?

The magnitude of PPH morbidity and mortality reveals the importance of its prevention and the identification of risk factors. Anemia and hypertensive syndromes stand out among its various risk factors. Risk stratification is a useful strategy for reducing maternal death from PPH. High-risk factors are: placenta previa or low-lying placenta, preeclampsia with signs of severity, hematocrit <30%, platelets <100,000/mm ^3^ , active bleeding on admission, coagulopathies, use of anticoagulants, placental abruption and placenta accreta. 1 6

The main preventive measures for PPH are the administration of oxytocin and the active management of the third stage. The most recommended prophylactic oxytocin regimen is the intramuscular administration of 10 units of oxytocin immediately after birth. In the case of cesarean section, an alternative is intravenous prophylaxis by the “rule of three”, in which three units of oxytocin are slowly infused and can be repeated at three-minute intervals until the third dose. This scheme should always be followed by maintenance intravenous infusion (15 units in 500 mL of 0.9% saline at 100 mL/hour). Active management of the third stage includes timely clamping (between one and three minutes) and controlled umbilical cord traction (Brandt-Andrews maneuver), skin-to-skin contact (for two hours or more) and uterine monitoring/massage in the first two hours after discharge. Other preventive measures include the rational use of oxytocin in labor, the selective use of episiotomy and the strict ban on Kristeller maneuver. 1 6

## How should the PPH diagnosis and the blood loss estimate be?

The most current definition establishes cumulative 1,000 mL as a diagnostic criterion for PPH, regardless of the mode of delivery. 1 However, especially if accompanied by risk factors, blood losses greater than 500 mL after vaginal deliveries should be considered abnormal. Losses above 1,000 mL are classified as severe PPH and greater than 2,000 mL as massive hemorrhage, usually accompanied by a hemoglobin drop ≥ 4 g/dL, coagulopathy and the need for massive transfusion. 10 Decreases in hematimetric indices (hemoglobin, hematocrit) are late and do not reflect the hematological state of the moment. They are representative of blood loss only about four hours after the onset of hemorrhage hence, clinically limited. 11 In addition, gestational hypervolemia delays the onset of the first signs of hypovolemic shock, especially among healthy pregnant women. In these, hemodynamic changes occur only after losses greater than 20% -30% of blood volume (1,500 to 2,000 mL). In view of the clinical evidence of blood loss above the usual, there must not be delays in the institution of treatment. 6

Strategies for diagnosing and estimating volume loss include visual estimates, weighing of compresses, collecting devices and clinical parameters, including the shock index.

**Figure 1.**
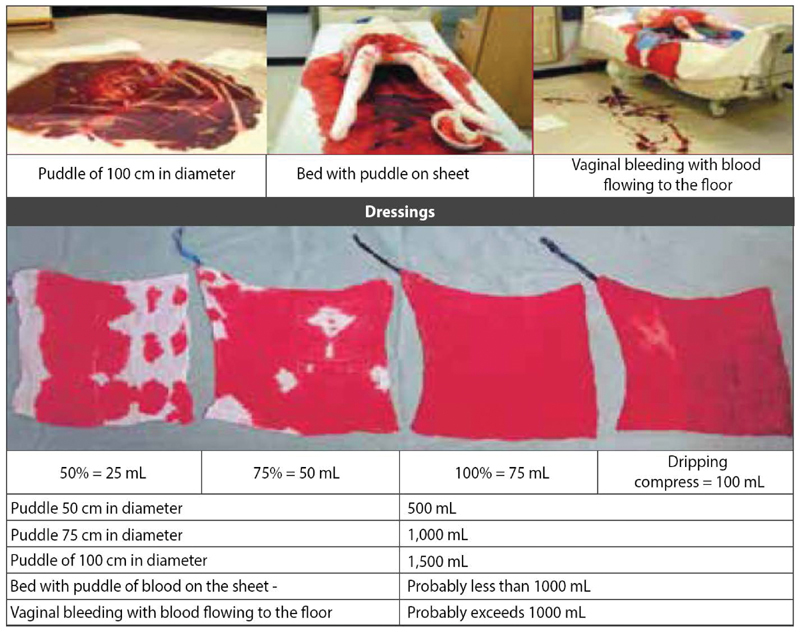
Visual parameters for quantifying the bleeding in surgical dressings, sheets and puddles. Source: Adapted from Bose P, Regan F, Paterson-Brown S. Improving the accuracy of estimated blood loss at obstetric haemorrhage using clinical reconstructions. BJOG.2006;113(8):919-24. 13 Dildy GA 3rd, Paine AR, George NC, Velasco C. Estimating blood loss: can teaching significantly improve visual estimation? Obstet Gynecol. 2004;104(3):601–6. 14

Although the visual estimate of blood loss is simple and quick, it is subjective and underestimates voluminous losses by up to 2-3 times. 12 Figure 1 shows some visual parameters for quantifying the bleeding present in surgical dressings, sheets and puddles.

Weighing of compresses, surgical packs, sheets and other supplies used in childbirth care is useful, especially in PPH linked to cesareans and hysterectomies. However, it requires knowledge and standardization of the size and weight of the inputs. By using the equivalent of 1 mL of blood and 1 g of weight, is obtained the blood loss in mL by calculating the difference between the weight of the blood-containing inputs and their dry weight. 6 15

The estimate with use of collecting devices positioned below the buttocks just after vaginal delivery is more reliable than the others, although still subject to failures, because of blood collection with the inclusion of amniotic fluid and urine. 6 16

Although clinical parameters (blood pressure, heart rate -HR) are late diagnostic markers, they are very useful for determining the severity of shock, evaluating the therapy instituted and indicating additional therapies. The shock index is an adjunct in the estimation of volume loss and an early marker of hemodynamic instability, with values that correlate with the need for blood transfusion and care transfer. Its calculation is made by dividing HR by systolic blood pressure (SBP). Values ≥ 0.9 indicate significant blood loss and ≥ 1 (HR higher than SBP) signal the need for a fast and aggressive approach and the possibility of blood transfusion. Values between 1.3 and 1.7 (moderate shock) and > 1.7 (severe shock) are indicative of assessing the need for massive transfusion. 17 18

## What should be the initial therapeutic measures in the PPH approach?

When PPH is diagnosed, the entire care team must know the steps of treatment according to the causes and be able to institute them. Defining the hemorrhagic etiology and estimating the severity of the condition are essential steps in care. The main causes of PPH are uterine atony, birth canal lacerations, placental disorders and coagulopathy (the four Ts: tone, trauma, tissue and thrombin), each requiring a specific approach. 1 6

**Figure 2.**
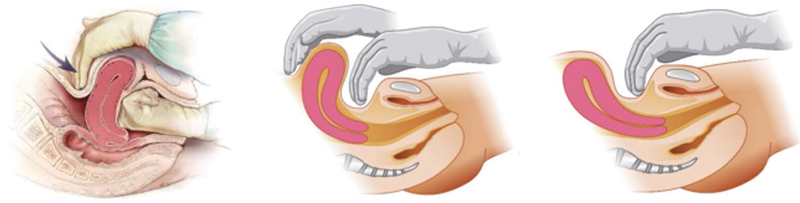
Hamilton and Chantrapitak uterine compression maneuvers. Source: Adapted from Anderson JM, Duncan E. Prevention and management of postpartum hemorrhage. Am Fam Physician. 2007;75(6):876-81. 20 Illustrations by Felipe Lage Starling (authorized). Left: Hamilton maneuver. Center: Chantrapitak maneuver for patients with relaxed abdominal wall. Right: Chantrapitak maneuver for patients with a tight abdominal wall.

Regardless of the cause, it is important that the entire team is familiar with the initial measures of care. The first step is to clearly communicate the diagnosis and organize the multidisciplinary team. The hemorrhage kit must be requested and one of the team members must be assigned for the communication and guidance of the patient and companions. Auxiliary professionals must know their roles and perform them simultaneously. A member should lead the team and ensure that actions are taken. In order to reduce bleeding, bimanual uterine compression is initiated by means of Hamilton maneuver (anesthetized patients or those with greater tolerability) or Chantrapitak ( Figure 2 ). 1 6 19 20

**Figure 3.**
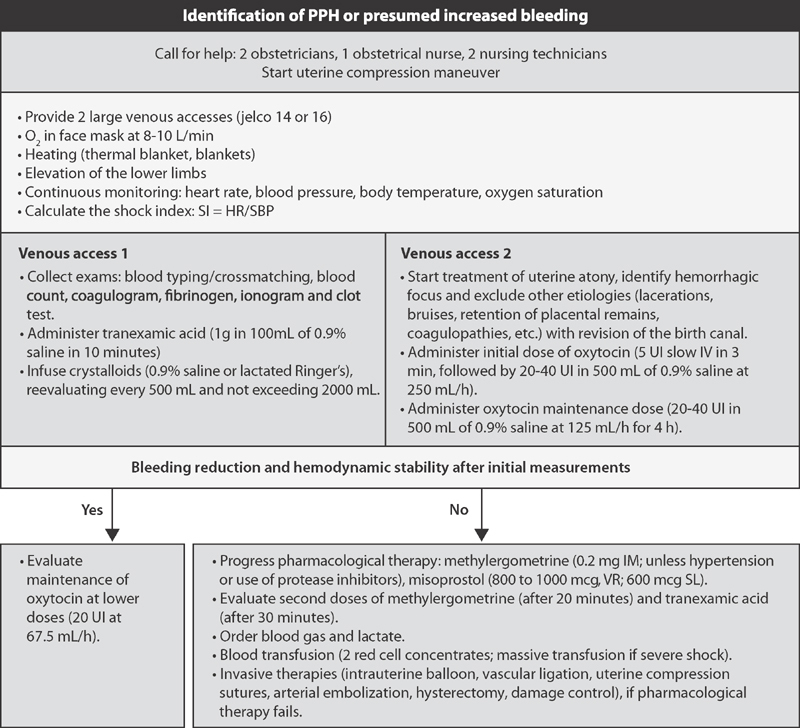
Sequencing of the initial clinical management of postpartum hemorrhage

An assistant must be responsible for the continuous monitoring of the patient to calculate the shock index. Two other assistants provide two large venous accesses (jelco 14 or 16), which will provide the infusion of crystalloids and medications and the collection of blood samples. Complementary tests should include blood typing (if unavailable), cross-matching, complete blood count, coagulogram, fibrinogen, ionogram, clot test (Wiener) and, in severe cases, lactate and blood gas analysis. Oxygenation with a face mask (100% O2; flow of 8 to 10 liters per minute) should be instituted. Indwelling urinary catheterization, elevation of the lower limbs, warming up of the puerperal woman, assessment of antibiotic prophylaxis, estimation of blood loss and rapid assessment of the etiology (revision of the birth canal), with location of hemorrhagic foci. Hemostatic measures should be instituted according to the etiology. When available, a non-pneumatic anti-shock garment (NASG) can be included in these initial measures. Subsequently, volume loss and hemodynamic repercussion are reassessed, with the intention of defining the need for blood transfusion. 6 Figure 3 systematizes the initial clinical management of PPH.

## How should the pharmacological management of PPH be?

Oxytocin and tranexamic acid should be the first drugs infused. Tranexamic acid should be administered at a dose of 1 gram diluted in 100 mL of 0.9% saline and can be repeated after 30 minutes if bleeding control has not been achieved. If bleeding recurs within 24 hours of initial administration, another dose of 1 gram can be infused. Uterotonic infusion regimens are variable. The initial slow infusion (three minutes) of 5 units of oxytocin is suggested, followed by 20 to 40 units in 500 ml of saline administered at 250 ml/hour. A sequential maintenance schedule should be administered at 125 mL/h for four hours. In the most severe cases of uterine atony, maintenance of oxytocin for up to 24 hours (67.5 mL/h or 3 units/h) should be considered with monitoring for water intoxication. 1 6 21

In view of an inadequate response to oxytocin, sequential infusion of other uterotonics is necessary and the time interval for decision making should not exceed 15 minutes, since these are fast-acting drugs. In the absence of arterial hypertension or the use of protease inhibitors, methylergometrine (0.2 mg intramuscular) should be the second uterotonic administered and may be repeated after 20 minutes. The last line uterotonic is prostaglandin. Rectal administration of 800 to 1,000 mcg of misoprostol or 600 mcg sublingually is suggested. 1 6

This drug sequencing is directed to uterine atony, the most frequent etiology of PPH. Parallel to its institution, the clot test and revision of the birth canal should be performed with the aim to exclude other etiologies (birth canal lacerations, uterine rupture or inversion, placental remains, coagulopathies). These etiologies require specific treatments, such as uterine curettage (placental remains), sutures (birth canal lacerations), uterine repositioning maneuver (uterine inversion), laparotomy for repair or hysterectomy (uterine rupture) and transfusion of blood components (coagulopathies). 1 6 Figure 3 shows the initial drug management of PPH.

## When and how to use an intrauterine balloon tamponade?

**Figure 4.**
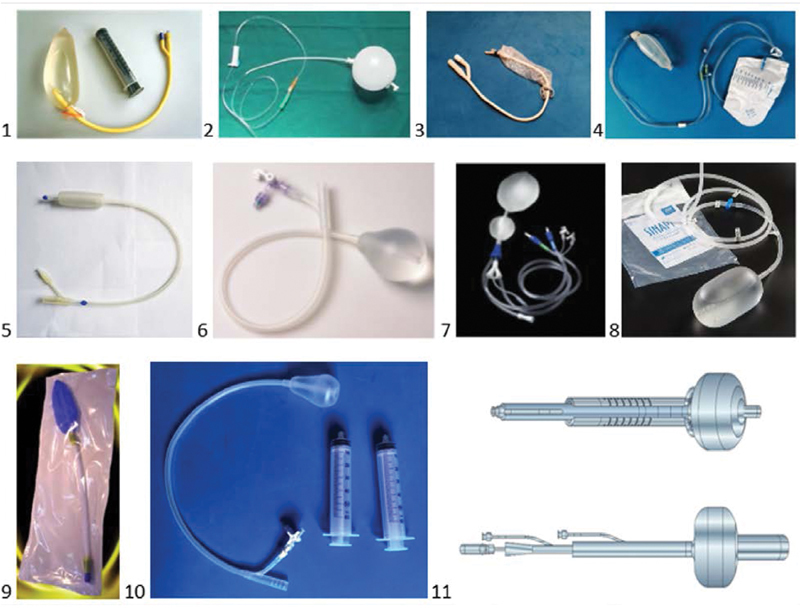
Intrauterine balloons. Source: photographic records of the authors; illustration by Felipe Lage Starling (authorized).Handcrafted: 1 – Shivkar; 2 – Baskett; 3 – ElHennawy; 4 – Alves; Manufactured: 5 – Bakri; 6 – BT-Cath; 7 – Ebb; 8 – Ellavi; 9 – Zhukovskiy; 10 – Pergo; 11 – Kyoto.

The main indication for an intrauterine balloon tamponade (UBT) is the failure of pharmacological therapy in uterine atony. As the achievement of transient hemostasis is also an objective of the tamponade, a balloon can be temporarily used in patients who will be transported to referral units or those with coagulopathy who need specific therapies. The main contraindications are pregnancy, infections in the internal genitalia, abnormalities distorting the uterine cavity, uterine rupture, allergy to balloon components and arterial bleeding requiring surgical treatment or embolization. 6 22 23 24 The UBT can be manufactured (Bakri, BT-Cath, Ebb, Zhukovskiy, Ellavi, Pergo, Kyoto) or, if these are unavailable, handcrafted (Shivkar, Baskett, El Menia, El Hennawy, Alves) ( Figure 4 ).

The UBT may or may not have a blood drainage function. The preparation for its postpartum vaginal insertion includes, in sequence, antisepsis (vulvar, vaginal and cervical), indwelling urinary catheter and inspection of the vagina and cervix. After clamping the anterior lip of the uterine cervix, the intrauterine balloon insertion can be performed manually or by means of Foerster forceps, guided or not by pelvic ultrasound. Before infusion, the balloon should be fixed, preferably with vaginal compresses. Alternatively, fixation can be performed by cervix suture or by apprehending the edges of the cervix with vascular clips or clamps. After fixation, the balloon is infused with saline. After vaginal delivery, the recommended infusion is between 350 and 500 mL. If a drainage system is available, its permeability must be checked (light infusion to ensure clearance) and connected to a collection bag. Antibiotic prophylaxis (cephalosporin) and maintenance dose oxytocin should be administered throughout the tamponade time. 6 22 23 25 26 27

After the infusion, the evaluation of the tamponade test begins. If within 30 minutes of infusion the drainage shows less than 50 mL, the prediction of successful tamponade is considered positive. Therefore, using balloons with a drainage function optimizes the tamponade test. 28

The balloons can be inserted during or after cesarean sections. In these situations, the infusion should be reduced (250 to 300 mL) in order to avoid dehiscence in hysterorrhaphy. When the balloon insertion is performed after the completed cesarean section, it is similar to the vaginal postpartum. The insertion of the balloon during cesarean section should be performed preferably by the abdominal route (via hysterotomy) and is more difficult in balloons with a three-way. This difficulty can be overcome by applying straps compressing the three-way and connecting a flexible probe to the balloon axis, adapting the probe as a guide so the balloon underpasses through the cervical canal. Another alternative is the introduction of a surgical forceps through the vaginal route into the uterine cavity. The balloon axis is grasped by the forceps and pulled into the vagina. If these strategies are not successful, the balloon will be inserted through the vaginal route. 6 25 29

The length of stay of the UBT can be up to 24 hours. However, in case of hemorrhagic control and hemodynamic stability, early removal is indicated. 30 Deflation in stages (100 mL every 15 minutes) is recommended, performed during the day with a reserved operating room, with maintenance oxytocin infusion. In case of hemorrhagic recurrence during the removal process, the UBT must be reinfused and the patient prepared for laparotomy. 23

## When and how to use NASG?

The NASG is a segmented neoprene garment that covers the lower limbs and abdomen from the ankle to the last rib, applying external compression. It is a low-cost, easy-to-use and washable device, and an adjunctive non-surgical intervention in volume resuscitation and in the treatment of severe forms of PPH. 31

**Figure 5.**
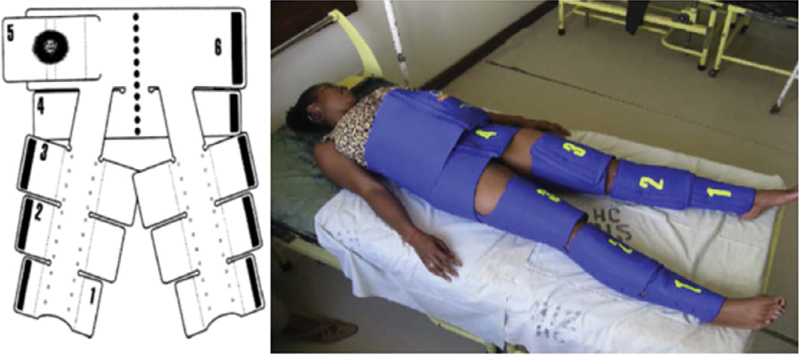
Non-pneumatic anti-shock garment. Source: Miller S, Martin HB, Morris JL. Anti-shock garment in postpartum haemorrhage. Best Pract Res Clin Obstet Gynaecol. 2008;22(6):1057–74. 31 ; https://lifewrap-nasg.com/

The NASG is divided into six articulated segments, three in each leg (numbers 1, 2 and 3; positioned just above the ankles, on the calves and on the thighs), one for the pelvis (number 4) and two for the abdomen (numbers 5 and 6). Segment 6 includes a foam compression ball that is positioned over the umbilical scar ( Figure 5 ). The well-fitted NASG applies a circumferential compression of 20 to 40 mmHg, forces blood flow to the upper limbs and central organs (brain, heart and lung) and provides increased blood pressure, preload and cardiac output. 31

The main benefit of NASG is reducing the speed of bleeding and the need for blood transfusion and additional surgery. Other advantages include the facilitation to obtain venous access, possibility of maintaining its use during perineal approaches (surgical and clinical) and laparotomy, and the gain of extra time for the etiological diagnosis, volume resuscitation, pharmacological treatment and patient transfers. 31 32

The NASG is indicated in case of bleeding with hemodynamic instability or imminent shock, and can be used in association with other invasive treatments (UBT, surgeries). Contraindications include a live viable fetus, severe heart disease (heart failure, mitral stenosis), severe respiratory disease (pulmonary hypertension, acute lung edema) and supradiaphragmatic injury. 31

Both placement and removal should be performed from segment 1 to 6 (from the ankle to abdomen), except in the case of laparotomy, in which the abdominal segments (5 and 6) can be removed in isolation. During the application, each segment must have its velcro well fitted and closed with adequate tension, keeping the joints (ankle, knee and hip) free. After placement, the NASG allows complete perineal access, thereby providing continuity of care (surgical procedures, uterine compression maneuvers). 6 32

The removal of the NASG must occur in hemodynamic stability and conditions to perform additional interventions. Blood loss <50 mL/hour, HR <100 bpm, SBP> 100 mmHg, hemoglobin (Hb) > 7 g/dL and hematocrit (Ht) > 20% are the recommended parameters for removal. The removal process must occur under hemodynamic monitoring, and the time interval between the removal of segments must be 20 minutes, allowing the redistribution of blood flow. The absence of a drop in SBP by 20 mmHg or a HR increase by 20 bpm is a parameter that can guide the progress of removal. The details of the removal process are justified by the possibility of a shock recurrence in inappropriate removals. In the event of recurrence of hemodynamic instability or bleeding, the NASG must be repositioned and additional treatments instituted. In the laparotomy situation, in which abdominal segments (5 and 6) are removed before the others, their removal must occur immediately before the procedure with an expected drop in blood pressure. 32

Although the length of stay of the NASG is not well defined yet, it is often between 6 and 8 hours. However, there are reports of its safe use for up to 48 to 72 hours. 6

## How should the hemostatic resuscitation management be?

It is estimated that 0.6% of deliveries require a hemotherapy approach motivated by hemorrhagic shock. 33 In this context, knowledge of the principles of hemostatic resuscitation and the institutional presence of a massive blood transfusion protocol, including emergency transfusion flow, are essential in birthing centers.

In addition to the rapid control of bleeding and the restoration of tissue perfusion, the aim of the strategy for hemorrhagic shock treatment is an early approach to coagulopathy and hypothermia. Temperatures below 35°C reduce tissue oxygen perfusion, favor acidosis and aggravate coagulopathy. 6 34

In volume resuscitation, the response should be assessed for every 500 mL of infused crystalloids. The rapid and excessive infusion of crystalloids can raise blood pressure before surgical control of the hemorrhagic focus, paradoxically increasing bleeding (destruction of formed clots), favoring hypothermia (unheated liquids) and diluting coagulation factors, which increases the risk of dilutional coagulopathy and progression to the deadly triad. After infusion of 1,500 mL of crystalloids, especially in the presence of active bleeding, hemodynamically unstable patients should be evaluated for immediate blood transfusion. After infusion of 2,000 mL of crystalloids, resuscitation should continue with blood components. 6 34

The shock index is useful in predicting the need for blood transfusion. As Hb and Ht measurements change late, they are not useful parameters in the initial management of hemostatic resuscitation. 6 17 18 34

Current transfusion protocols are varied and based on trauma studies. However, as the evolution to hypofibrinogenemia is earlier in PPH, this is an important aspect to be considered in hemostatic resuscitation. Fibrinogen levels below 200 mg/dL have a 100% positive predictive value for severe PPH. Therefore, an aggressive approach to hypofibrinogenemia is essential. 35

The initial transfusion decision should be based on the patient's clinical status (shock index). The proportions of the use of blood components and the transfusion goals must be included in the protocols. Hemostatic resuscitation is usually necessary in patients with no clinical response to initial volume replacement with crystalloids. Hemodynamically unstable patients with significant losses should receive emergency transfusion of two red cell concentrates. If crossmatching is not available, O negative blood should be transfused. In mild shock (SI ≥ 1), blood transfusion is usually not necessary and, if it occurs, it must be performed with compatible typed blood. In the face of severe shock (SI> 1.7), transfusion must be massive and performed with equal proportions of red cell concentrate, fresh frozen plasma, cryoprecipitate and platelets. Fibrinogen must be measured and, when available, viscoelastic tests may contribute to reduce the use of blood components. Therapeutic targets are Hb> 8 g/dL, fibrinogen between 150 and 200 mg/dL, platelets> 50,000/mm3 and INR ≤ 1.5. 6 34

## Final considerations

Since PPH is the major cause of maternal mortality in the world, it is essential that care teams are able to prevent, diagnose and institute non-surgical management within the “golden hour”. The need for simultaneous institution of multiple actions for the adequate therapeutic management of PPH justifies the presence of an orderly work system in care centers. For the reduction of risks and morbidity and mortality from PPH, it is necessary to implement risk stratification in health services and reduce difficulties in the management of patients by the early identification of risk factors and optimization of prenatal, childbirth and post-natal care. The systematic use of prophylactic oxytocin and active management of the third stage and an efficient method of estimating blood loss, combined with the appropriate diagnosis and therapeutic are practices that should be offered in a standardized and uniform manner by care teams. The availability of UBT, NASG and blood components and the knowledge and skill of professionals for the correct use of these inputs complement the care needs for an adequate non-surgical management of PPH. Finally, the valorization of women's lives, the organization of local health systems and the establishment of programs dedicated to preventing maternal mortality that improve health professionals' skills and eliminate barriers to care access are essentially the greatest challenges to reduce PPH morbidity and mortality.

National Specialty Commission for Obstetric Emergencies of the Brazilian Federation of Gynecology and Obstetrics Associations (FEBRASGO)

President:

Álvaro Luiz Lage Alves

Members:

Gabriel Costa Osanan

Samira El Maerrawi Tebecherane Haddad

Adriana Amorim Francisco

Alexandre Massao Nozaki

Brena Carvalho Pinto de Melo

Breno José Acauan Filho

Carla Betina Andreucci Polido

Eduardo Cordioli

Frederico José Amedée Peret

Gilberto Nagahama

Laíses Braga Vieira

Lucas Barbosa da Silva

Marcelo Guimarães Rodrigues
